# Coactivator networks orchestrating noncanonical AR programs in enzalutamide-resistant CRPC

**DOI:** 10.3389/fonc.2025.1748527

**Published:** 2026-01-12

**Authors:** Ephraim J. Gardner, Sasikumar Ponnusamy, Remi Adelaiye-Ogala

**Affiliations:** 1 Division of Hematology and Oncology, Department of Medicine, Jacobs School of Medicine and Biomedical Sciences, University at Buffalo, Buffalo, NY, United States; 2 Department of Pharmacology and Toxicology, Jacobs School of Medicine and Biomedical Sciences, University at Buffalo, Buffalo, NY, United States; 3 Department of Genetics, Genomics and Bioinformatics, Jacobs School of Medicine and Biomedical Sciences, University at Buffalo, Buffalo, NY, United States; 4 Department of Urology, Jacobs School of Medicine and Biomedical Sciences, University at Buffalo, Buffalo, NY, United States

**Keywords:** advanced prostate cancer, androgen receptor, AR cistrome, canonical and noncanonical AR cistrome, enzalutamide resistance, lineage plasticity, noncanonical AR coactivator, therapeutic strategies

## Abstract

Resistance to androgen receptor (AR)-targeted therapies remains a major clinical challenge in the treatment of castration-resistant prostate cancer (CRPC). Emerging evidence suggests that Enzalutamide resistance is not solely due to the loss of AR dependency but can also arise from epigenomic reprogramming of the AR cistrome toward noncanonical gene networks. Recent studies have revealed that this reprogramming is mediated by previously unrecognized coactivators, including CXXC5, TET2, and EZH2, which cooperate with AR to establish a transcriptional landscape that supports lineage plasticity and therapeutic evasion. These noncanonical AR transcriptional programs enable CRPC cells to survive under continued AR blockade, acting as a transitional state towards neuroendocrine differentiation. Pharmacologic disruption of these coactivators abrogates noncanonical AR activity and suppresses tumor growth, highlighting a tractable vulnerability. These findings redefine AR signaling in advanced disease, suggesting that targeting noncanonical AR coactivators could offer a novel therapeutic paradigm to overcome resistance. Advances in single-cell and epigenomic profiling are poised to delineate further the heterogeneity and dynamics of AR cistrome remodeling in treatment-refractory prostate cancer.

## 1 Introduction

The androgen receptor (AR) is essential for the growth and development of the prostate (1, 2). AR is a member of the steroid hormone nuclear receptor family and canonically requires androgen binding, along with the recruitment of cofactors, to regulate the transcription of target genes (1). While AR-mediated transcription is required for male sexual differentiation, aberrant androgen-driven AR activity is a crucial driver of prostate cancer (PCa) formation and progression (1, 2). Thus, first-line treatment of advanced PCa aims to deplete androgen levels in patients via surgical or chemical castration. This typically leads to disease remission, though castration-resistant prostate cancer (CRPC) inevitably arises in patients (1, 2). CRPC treatment relies on direct targeting of AR via signaling inhibitors such as Enzalutamide, Apalutamide, and Darolutamide, which bind the ligand-binding domain (LBD) of AR and prevent its nuclear translocation. However, multiple mechanisms of resistance, specifically, Enzalutamide resistance (Enza-R) have been elucidated, such as reactivation of canonical AR signaling (3–5), generation of AR splice variants lacking the ligand binding domain (3–5), mutation of the AR (3–5), and upregulation of alternative signaling pathways such as glucocorticoid receptor (GR)-dependent signaling (3–6).

Another emerging mechanism of treatment resistance involves reprogramming of the AR cistrome, altering canonical AR signaling, and enabling AR binding to novel noncanonical genomic sites. A cistrome refers to the cis-regulatory elements (ex., binding motifs) on a strand of DNA and the trans-acting factors (ex., transcription factors) that bind at these elements to mediate transcription of relatively close or distal genes via binding at promoters or enhancers, respectively (7). Canonical AR signaling consists of AR binding via its DNA binding domain (DBD) to androgen response elements (ARE) at promoters or enhancers of target genes that are widely known to be essential in prostate growth and development, as well as in PCa progression, such as *KLK2, KLK3 (*PSA*)*, *NKX3.1*, *TMPRSS2*, *FKBP5*, and others (8, 9). However, studies have shown that the AR cistrome is heavily reprogrammed during the transition from a normal prostate to primary PCa and from primary PCa to CRPC (10, 11). Specifically, AR cistrome reprogramming from primary PCa to metastatic CRPC (mCRPC) resulted in a transcriptional state reversal toward embryonic prostate development, suggesting a role for AR cistrome reprogramming in PCa lineage plasticity (11). Additionally, a noncanonical AR cistrome signature has been found to be associated with disease progression, metastasis, and poorer prognosis for PCa patients (12).

**Figure 1:**
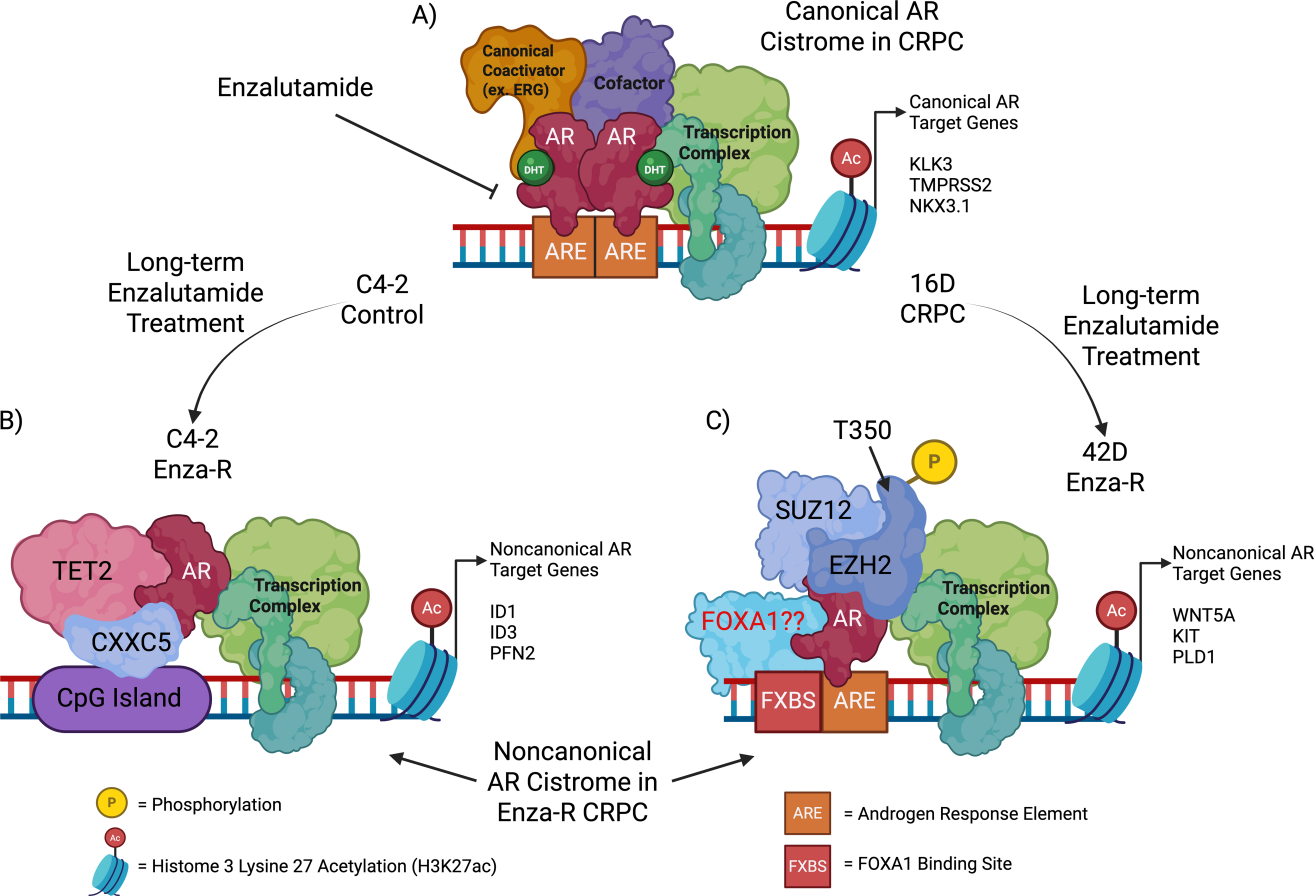
AR cistrome reprogramming in Enzalutamide-resistant-CRPC. **(A)** AR is bound to androgen response elements (ARE) at transcriptionally active canonical genomic loci, where it complexes with coactivators to drive the expression of canonical target genes that allow for proliferation and survival of PCa. Enzalutamide directly targets AR, leading to a loss in canonical AR cistrome binding and activation of Enzalutamide resistance mechanisms. **(B)** A model of noncanonical AR cistrome reprogramming identifying CXXC5 and TET2 as noncanonical coactivators of AR. These findings were discovered in C4–2 Enza-R cell lines as compared to the CRPC control. **(C)** A model of noncanonical AR cistrome reprogramming identifying EZH2 as a noncanonical coactivator of AR. SUZ12 was also found to be part of the noncanonical binding complex, though AR and EZH2 are the definitive drivers of the noncanonical AR cistrome. These findings were observed in 42D Enza-R cells compared with the CRPC control. Abstract created using BioRender.

In recent years, many studies have increased our understanding of the mechanisms underlying AR cistrome reprogramming. However, this review will focus on the mechanisms underlying this switch in response to the selective pressures of AR-targeted therapies such as Enzalutamide. The review will discuss known AR cistrome modulators and cover novel AR cofactors identified with Enza-R, as well as noncanonical AR cistrome reprogramming. The downstream consequences of these resistance pathways result in altered, noncanonical AR-dependent gene expression in PCa, contributing to transitional lineage plasticity and a neuroendocrine state of PCa. A schematic representation of AR cistrome reprogramming in Enzalutamide-resistant-CRPC is shown in **Figure 1**.

## 2 Literature review methodology

The literature for this review was collected using a comprehensive search strategy across reputable scientific databases, including the National Center for Biotechnology Information (NCBI) PubMed and Google Scholar. The inclusion criteria were as follows: full-text articles and notable peer-reviewed abstracts published through 2025 and in English. Full-text unavailable articles, non-English language, or summaries of editorials, conferences, or seminars were excluded. Keywords such as advanced prostate cancer, androgen receptor, AR cistrome, canonical and non-canonical AR cistrome, lineage plasticity, therapeutic strategies, Enzalutamide resistant prostate cancer, noncanonical AR cistrome reprogramming, and AR-dependent and independent gene expression were used alone or in combination.

## 3 Discovery of AR cofactors mediating treatment resistance

As a master transcription factor, AR is known to interact with a diverse array of proteins to carry out its transcriptional activities. Chromatin remodeling complexes and chromatin readers, like the SWI/SNF complexes and BET protein family, are known to associate with AR and AR cofactors to drive aggressive disease (7). Several pioneer and transcription factors, such as FOXA1, HOXB13, GATA2, and ERG, have previously been identified with AR cistrome reprogramming in early and progressive stages of the disease (primary and CRPC) (10, 11), their roles having been extensively covered by several other reviews (7, 13–16). Other coactivators, such as CBP/p300, play crucial roles in promoting AR genomic accessibility and transcriptional activity as PCa progresses (1, 17). More recently, in Enzalutamide-resistant CRPC (Enza-R CRPC), research groups have uncovered proteins previously unknown to interact with AR at the epigenomic level within the cell, and identified known AR coactivators that mediate a noncanonical AR switch. These new findings and their implications for noncanonical AR cistrome reprogramming will be elaborated on further below.

### 3.1 SWI/SNF complex and BET proteins: master chromatin remodelers that facilitate oncogenic AR activity

The Switch Sucrose Non-Fermenting (SWI/SNF) complexes, also known as Brg/Brahma-associated factor (BAF) complexes, consist of 29 protein subunits that coalesce into three well studied chromatin remodeling complexes (18, 19). These complexes facilitate chromatin accessibility by repositioning nucleosomes through sliding or removal actions (19, 20); these actions are powered by two ATPase subunits, SMARCA4 (BRG1) and SMARCA2 (BRM), that bind to the SWI/SNF complexes in a mutually exclusive manner (7, 19). SWI/SNF complexes are known to play key roles in determination of cell fate during embryogenesis (18, 21), and they are characterized as controlling dynamic gene expression via distal enhancer or proximal promoter regions (21). Importantly, mutations occur in SWI/SNF subunits in over 20 percent of human cancers (18, 19, 22), and loss of function mutations, along with varied expression of SWI/SNF proteins, contribute to the perturbation of SWI/SNF complex in a nuanced manner (19, 21, 23). Such perturbations and their impacts in cancer settings have been extensively covered in other reviews (19, 21, 23). In prostate cancer, SMARCA4 expression has been positively correlated with disease progression through IHC of patient tumor samples, with the highest levels observed in NEPC (18); the opposite trend was seen with SMARCA2 expression (18). Interestingly, an enrichment of neural SWI/SNF subunit genes were identified at the NEPC stage, implicating SWI/SNF as a modulator of lineage plasticity via expression of specific subunits (18). In the context of the SWI/SNF complex transcriptional role in prostate cancer, core subunits of the SWI/SNF complex have been found to physically interact with AR and AR cofactors FOXA1 and ERG (7, 22). These interactions were enforced by ChIP-seq studies in VCaP and LNCaP cell lines, which revealed that SWI/SNF complexes modulate AR, FOXA1, and ERG cistrome binding by increasing chromatin accessibility at H3K27ac enriched-distal enhancer regions (22). Of note, SWI/SNF complexes are known to recruit p300 (21), a histone acetyltransferase, while SWI/SNF complexes themselves are recruited to compact chromatin by pioneer factors (21); these findings may help explain how the SWI/SNF-AR-FOXA1-ERG core enhancer circuitry was established in PCa (22). Ablation of both ATPase subunits, SMARCA2 and SMARCA4, by the PROTAC degrader AU-15300, resulted in collapse of the enhancer circuitry, as evidenced by loss of chromatin accessibility and looping interactions, depleted H3K27ac signal and binding of AR, FOXA1, and ERG at enhancer regions, subsequent loss of target gene expression for those TFs, and reduced expression of AR, FOXA1, and ERG (22). While SWI/SNF ATPase degradation also resulted in reduced proliferation of Enza-R cell lines compared to parental cell lines (22), suggesting that SWI/SNF may be involved in AR-dependent activity in Enzalutamide resistance, a recent study provided counter evidence by showing an additive, but not a synergistic, effect in Enza-R cell lines treated with BAF inhibitors or degraders in combination with Enzalutamide (24). This study, which utilized a CRISPR screen on all epigenetic enzymes in LNCaP cells and its Enza-R derivatives, identified the SWI/SNF subunits SMARCC2 and DPF2 as being essential to Enza-R cell lines (24). Further epigenetic findings revealed SMARCC2 gained binding sites in an Enza-R cell line (MR49F) that were alone or overlapped with BRG1 (SMARCA4) (24); these novel sites (BAF-Enza) were enriched for loci of MYC, ETS factors, MAPK1, and transcription machinery factors, while conserved (BAF-Adeno) sites were enriched for AR, FOXA1, and other AR-cofactors (24). From these collective findings, the authors suggest that the SWI/SNF complex acts independently of AR at the Enza-R disease stage. However, the Enza-R cell line models utilized in this study (24) and the previous study (22) were cultured in androgen-rich FBS media despite undergoing constant Enzalutamide treatment; the residual androgens present in these culture conditions may modulate AR activity in a way that does not fully recapture the Enza-R CRPC disease stage. Furthermore, AR binding at SMARCC2/BRG1 sites was determined using publicly available datasets; an AR ChIP-seq performed in this setting may have revealed non-canonical AR involvement at the gained SWI/SNF sites. Nonetheless, the SWI/SNF complex has been implicated in prostate cancer disease progression and has been shown to play a key role in the modulation of the AR cistrome.

The Bromo- and Extra-Terminal (BET) protein family (comprised of BRD2, BRD3, BRD4, and BRDT) are a group of widely expressed chromatin readers that recognize acetylated lysine on histones and recruit transcription factors and transcriptional regulatory complexes to accessible genomic sites (25–27). The “reader” activity is driven by two bromodomains, BD1 and BD2, which are highly conserved in these proteins (25–27). BET proteins, namely BRD4, are recruited to super enhancers, where they are densely bound along with other transcription factors, mediators, and transcriptional machinery (26, 28); these transcriptional hubs regulate and drive oncogenic gene expression in many cancer types (26, 28). BRD4 has been associated with various oncogenic transcription factors, including being co-implicated with the estrogen receptor in breast cancer and with the androgen receptor in prostate cancer (27–29). In AR-positive PCa cell lines, BET inhibition with the dual BD1/BD2-targeting compund JQ1 resulted in a decrease in canonical AR target genes, including ERG expression, and a diminished AR gene signature, subsequently inhibiting growth in these models (27). The impact of JQ1 on proliferation was recapitulated in *in-vivo* CRPC xenograft models, with greater efficacy than Enzalutamide (27). AR was found to physically interact with BRD2-4, with a specific interaction occurring between the BD1/BD2 bromodomains and the NTD of AR (27). BET inhibition was shown to attenuate BRD4 and AR binding at co-occupied super enhancers and promoters of AR target genes (27). In a subsequent study, BET inhibition reduced proliferation of Enza-R xenograft-derived cell lines with active canonical AR signaling (30), while simultaneously decreasing AR-v7, but not AR, protein levels (30). Furthermore, in an *in-vivo* castrated VCaP xenograft model, combined treatment with JQ1 and Enzalutamide led to an enhanced antitumor effect, with depletion of *AR-v7, ERG*, and *MYC* transcript levels observed with solo JQ1 treatment and combination treatment (30). In a more recent study, a selective BD2 inhibitor named ABBV-744 displayed profound antiproliferative activity against prostate cancer cell lines that expressed AR, but not the splice variant AR-v7 (31). In LNCaP cells, ABBV-744 had a more targeted depletion of AR transcription in comparison to the dual BD1/BD2 inhibitor ABBV-075, which had wider impacts on global transcription (31). ABBV-744 was also effective in LNCaP at abrogating binding at co-bound AR and BRD4 super enhancers, which were enriched compared to non-AR bound super enhancers, though it was ineffective in AR-v7-expressing 22Rv1 cells (31). This finding provides additional evidence that AR directly interacts with both the BD1 and BD2 domains of BRD4 (27), and this interaction is nullified by the loss of the K_630_LKK_633_ motif in AR-v7 (31); interestingly, the broader effects of dual BD1/BD2 inhibitors such as JQ1 impact the expression of splicing factors, resulting in a decrease in AR-v7 production and reduced proliferation in Enza-R modelsthat express AR-v7 (30). Another recent study identified BRD4 as having a lesser impact on the AR transcriptome in CRPC; despite BRD4 and AR having co-bound loci, BET inhibition did not decrease androgen-dependent AR target gene signatures, but proved more effective in depleting pathways driven by gastrointestinal transcription factors HNF4G and HNF1A. Overall, BET family proteins, particularly BRD4, have been implicated as critical drivers of prostate cancer in concert with AR across stages of disease, with the BRD4-AR axis being increasingly targeted in preclinical settings and in clinical trials (28).

As mentioned above, many compounds have been developed to target bromodomain proteins, including members of the BET family and the ATPase subunits of the SWI/SNF complex. However, due to high structural similarity of bromodomains and a prevalence of bromodomain proteins in healthy tissues, toxicity has been a roadblock in the progression of these drugs into the clinic (31, 32). Development of more targeted inhibitors, such as the aforementioned ABBV-744 (31) and PROTAC degraders (22), which mark their targets for proteasome-mediated destruction, may be able to reduce treatment-related toxicity. Preclinical and clinical findings on improved bromodomain inhibitors and PROTAC degraders of BET family proteins and SWI/SNF ATPases, as well as other therapeutic targets for SWI/SNF subunits, have been thoroughly discussed in recent reviews (21, 23, 28, 33).

### 3.2 FOXA1, HOXB13, and GATA2: pioneer factors remodeling the AR cistrome through disease progression

Pioneer factors are unique transcription factors that can bind to DNA motifs in condensed chromatin regions, recruiting chromatin remodelers to transform the locus into an accessible stretch of the genome, allowing for downstream transcriptional activity to occur. Thus, these proteins are known as “pioneers” due to their ability to pre-mark loci and facilitate binding for other transcription factors, highlighting a crucial role in dictating cistrome activity and cell identity or cell fate (7, 13). FOXA1, HOXB13, and GATA2 are all pioneer factors of AR that are heavily involved in normal prostate development, and their binding patterns and cooperation vary as prostate cancer progresses.

FOXA1 (Forkhead box A1) is a member of the FOX protein family, which all contain a forkhead domain that allows for a single FOX protein to interact with DNA at a consensus motif (13). Studies have shown that FOXA1 displays pioneering activity with other members of the steroid nuclear hormone receptor family, such as the glucocorticoid receptor (GR) and the estrogen receptor (ER) (13). FOXA1 physically interacts with AR (13), and they cooperate to drive normal prostate growth by allowing FOXA1 to open access to AR at enhancers of androgen target genes (7). FOXA1 plays a pivotal role in prostate cancer progression as its upregulation in a normal immortalized prostate epithelial cell line (LHSAR) resulted in reprogramming of the AR cistrome towards a state seen in primary prostate tumors (10). Additionally, FOXA1 was discovered to bind the genome at sites in normal prostate that AR would bind later in primary PCa, and in primary PCa tissues that AR would bind later in mCRPC (11). These findings give credence to FOXA1’s pioneering role in directing AR towards pre-marked genomic sites crucial to the onset of malignancy and the survival of tumor cells as therapeutic pressures mount. Interestingly, FOXA1 is one of the most frequently mutated genes in prostate cancer patients: numerous specific coding and non-coding mutations have been highlighted in other reviews, with each alteration having diverse consequences on the AR cistrome (7, 13, 14). FOXA1 mutations from a cohort of over 1500 patients have been classified into three distinct structural classes to better understand their function in the initiation and progression of prostate cancer (34). A follow-up study, which resulted in the generation of mice bearing prostate-specific Class 1 and Class 2 FOXA1 mutations, revealed that Class 1 mutations (in a p53-deletion background), which occur in the DNA-binding forkhead domain and increase FOXA1 transactivation potential (34), result in spontaneous initiation of adenocarcinoma that becomes more aggressive as the mice age (35). This transformation is driven by an upregulation in AR and PI3K-mTORC pathways, which resulted in sensitivity of those tumors to castration; further epigenetic investigation uncovered AR cistrome reprogramming towards hARE: FOX sites in an NSD2-dependent manner (35). Conversely, Class 2 mutations, which involve frameshift mutations in the C-terminal domain and result in stronger chromatin binding and activation of WNT signaling, were unable to initiate tumor growth but did lead to differentiation of the prostate luminal epithelia (35). From this differentiation arose luminal stem-like cells, which were driven by KLF5/AP1 activity at FOXA1-reprogrammed loci lacking AR motifs (35). Organoids derived from these cells were resistant to castration and Enzalutamide treatment, and were able to form allografts in normal mouse prostate as opposed to Class 1-derived organoids (35). Overall, FOXA1 mutations have been shown to mediate tumor initiation or to potentiate therapeutic resistance via lineage plasticity, with these processes occurring either independently or in dependence on AR.

HOXB13 belongs to the Hox subgroup of homeobox DNA-binding domain proteins, known to be key regulators of vertebrate development (13). Specifically, HOXB13 is expressed in the developing prostate, where it drives luminal epithelial differentiation, and is stably expressed in the mature prostate during adulthood (13). While HOXB13 is not heavily mutated in PCa, its expression is upregulated in the primary stage of disease. Like FOXA1, HOXB13 can physically interact with AR and colocalize with it in shared target regions, thereby enhancing expression of those genes (13). HOXB13 can also influence AR gene expression, which may further modulate AR cistrome patterns (16). Along with FOXA1, HOXB13 overexpression in LHSAR cells contributed to AR cistrome reprogramming towards a primary tumor-patterned state (10). Interestingly, combined overexpression of FOXA1 and HOXB13 in the normal prostate model LHSAR resulted in the most significant transformation of the AR cistrome towards noncanonical binding sites observed in primary malignancy, with FOXA1 and HOXB13 showing extensive overlap at these primary tumor AR binding sites (10). As previously mentioned for FOXA1, the same study found that HOXB13 also pre-marked noncanonical genomic loci in the normal adult prostate and primary tumor stages (7), and that AR subsequently bound these loci in the following primary tumor and mCRPC stages, respectively (11). Remarkably, the cistromes of both FOXA1 and HOXB13 remained largely unchanged between the primary PCa and mCRPC stages of disease, again suggesting that both pioneer factors keep noncanonical loci accessible to AR for future binding as the disease progresses into castration resistance and metastasis (11). At the CRPC disease stage, HOXB13 has been found instrumental in dictating the cistrome reprogramming of AR-v7, a well-studied splice variant of AR that lacks a ligand-binding domain, is constitutively active, and develops as a resistance mechanism to androgen deprivation therapy (7, 13, 36). A recent study investigating an AR enhancer identified in LNCaP cells, which has a chromatin profile consistent with CRPC disease, revealed binding of HOXB13 and GATA2 to the enhancer in patient-derived xenograft (PDX) models and in an Enza-R CRPC cell line expressing AR-v7 (37). Thus, a novel role for HOXB13 and GATA2 in regulating AR expression in advanced PCa disease has been uncovered. HOXB13 depletion was also linked to reprogramming of the FOXA1 cistrome, including reducing FOXA1 binding at the AR enhancer, suggesting HOXB13 can modulate the activity of another pioneer factor (37).

GATA2 (GATA binding protein 2) is part of the GATA family of zinc finger transcription factors, which display pioneer activity; GATA2 has been shown to direct developmental programs in hematopoietic cell lineages and is expressed in urogenital tissues (7, 13, 38). GATA2 can increase genomic accessibility to AR by recruiting p300, an acetyltransferase, and it binds to enhancers of AR target regions prior to AR binding (7). In hormone-sensitive LNCaP cells, GATA2 was found to overlap with FOXA1 at more than 50% of sites where AR would later bind (7, 13). Whereas FOXA1 and HOXB13 function to direct AR towards noncanonical binding sites through the progression of disease stages, GATA2 further enhances canonical AR binding. Like HOXB13, GATA2 can also regulate AR gene expression (16), possibly contributing to an additional impact on the AR cistrome. In CRPC, GATA2 expression is upregulated, with higher levels correlating with more aggressive tumors and with chemotherapy in CRPC patients (7, 13). At this stage, GATA2 continues to direct the AR cistrome towards canonical target genes, even as Enzalutamide is introduced to antagonize AR signaling directly (7, 13).

FOXA1, HOXB13, and GATA2 are pioneer factors critical to prostate development, but they also act as oncogenes in prostate cancer. These pioneer factors contribute to aberrant AR activity by, often in concert, reprogramming the AR cistrome as the cancer becomes more advanced and aggressive. Whether each of these potent chromatin remodelers plays a crucial role in AR cistrome reprogramming in CRPC at the stage of Enzalutamide resistance remains to be seen. However, evidence for FOXA1’s involvement will be discussed in later sections of this review.

### 3.3 CBP/p300 and ERG: key AR coactivators and modulators of AR activity

The histone acetyltransferases (HATs) CBP (cAMP response element-binding protein (CREB)) and p300 are functional homologs that play important roles in transcriptional regulation and activation (17, 39, 40). Both proteins are promiscuous and physically interact with a plethora of proteins and transcription factors, including at the N-terminal domain of AR (40–42). CBP/p300 increases genomic accessibility through its HAT activity, allowing transcription factors to bind and modulate gene expression (17, 40). CBP/p300 also physically interacts with basal transcription factors, such as the TATA-binding protein and RNA polymerase II, providing additional structural support for an active transcriptional complex (17, 40). CBP/p300 have been studied extensively as coactivators of AR, and they are directly associated with the progression of prostate cancer (17, 40). CBP/p300 are recruited once AR binds to its DNA target sequence: CBP has been found to stimulate AR transcriptional activity in LNCaP cells (43), while p300 was discovered to enhance AR activity by acetylating an RXKK motif located proximal to the AR DNA binding domain at the C-terminal end (40). Though CBP/p300 are ubiquitously expressed across tissues, they were found to be upregulated at the gene and protein level in both primary and mCRPC samples from PCa patients, and their expression positively correlated with AR expression (17, 41). In a cell line model of the CRPC disease stage, p300 was found to be the predominant transcriptional activator of AR, leading to increased histone acetylation at transcription start sites (TSS) and AREs of AR target genes following androgen stimulation (41). A recent study reported the first solved electron cryo-microscopy (cryo-EM) 3D structure of full-length, R1881-bound AR, which binds to ARE DNA as a homodimer in a head-to-head, tail-to-tail arrangement (44). A subsequently solved structure, rendered after the addition of p300 and SRC-3 (an AR coactivator), included full-length AR homodimers bound to ARE DNA in complex with one p300 molecule and one SRC-3 molecule (44). While p300 was shown to bind SRC-3, it was also directly bound to the N-terminal domain (NTD) regions of the AR homodimer, as previously identified (40–42, 44). Importantly, the solved complexes were found to be functionally active in an *in-vitro* assay to measure transcription of a reporter gene, with the presence of SRC-3 and p300 greatly increasing AR transactivation (44).

ERG (ETS-related gene) hails from the family of E-26 transformation-specific (ETS) transcription factors (45). Studies using embryonic mice have revealed that ERG plays an integral role in maintaining vascular stability and integrity during development (45–47). ERG was also found to be necessary for both definitive hematopoiesis during embryogenesis and the control of hematopoietic stem cell function in adulthood (45, 48). However, aberrant ERG expression and activity have resulted in its identification as an oncogene, contributing to the progression of diseases such as Ewing’s sarcoma, acute myeloid leukemia, and prostate cancer (7, 45, 48). While ERG is not normally expressed in the adult human prostate epithelia (49), ERG overexpression in prostate cancer is fueled by the fusion of the *ERG* gene to the promoter regions of AR target genes (7, 45, 48). A prominent occurrence is the *TMPRSS2:ERG* fusion, which occurs in about half of primary prostate cancer patients (7, 49). Higher ERG expression levels are associated with more aggressive primary tumor stage, and ERG overexpression has been linked to the transition of localized tumor towards metastasis (45). Upon AR-regulated ERG overexpression within the prostate, ERG has been shown to interact with AR in various ways to modulate the AR cistrome and influence AR transcriptional activity (7, 45). A study that performed H3K27ac ChIP-seq on primary PCa patient tumors with or without *TMPRSS2:ERG* fusion revealed global remodeling of cis-regulatory elements (7, 50). The novel accessible sites upregulated in *TMPRSS2:ERG* fusion patients contained enriched DNA-binding motifs for ERG, AR, and the pioneer factors FOXA1 and HOXB13 (50). The upregulated sites were further validated in *TMPRSS2:ERG* positive VCaP cells, where ERG, AR, FOXA1, and HOXB13 were found to bind at these sites physically (50). The activity of ERG seems to be heavily tied to the genetic background of the models used in several studies. In VCaP cells, which are PTEN-positive, ERG enhances AR occupancy at co-bound sites with increased DHT exposure. Still, ERG also appears to repress transcriptional output of AR canonical targets such as *KLK3* (PSA) and *FKBP5* (51). In this setting, ERG acts as an AR corepressor, inhibiting AR epithelial programs and favoring a dedifferentiated tumor state that enables metastasis (45, 51). However, many patients with *TMPRSS2:ERG* fusion at both the primary PCa and CRPC stage simultaneously have *PTEN* and *TP53* alterations (52), and several studies have investigated ERG’s role in this genetic background. Utilizing a mouse model with *PTEN* deletion, *TP53* mutation, and prostate-specific *ERG* overexpression, a study by Blee et al. (2018) uncovered a role for ERG in preserving AR transcriptional activity and a luminal epithelial lineage program while simultaneously downregulating mesenchymal and neuroendocrine genes (52). In this setting, ERG limited the lineage plasticity induced by *PTEN* and *TP53* alterations (52). At the same time, its protection of the canonical AR cistrome also led to greater sensitivity to Enzalutamide treatment than *PTEN* deletion, *TP53* mutation, and *ERG* null models (52). Chen et al. (2013) generated a mouse model with *PTEN* deletion and prostate-specific *ERG* overexpression, resulting in highly invasive and aggressive tumors (53). In this setting, ERG was shown to reprogram the AR cistrome (while not altering AR expression) by increasing AR binding at conserved and novel sites (53). Interestingly, ERG appeared to function like a pioneer factor in this setting by promoting AR binding at sites with previously established H3K4me1 enhancer signal (53); while only 40% of novel AR sites were co-bound by ERG, almost 80% of the novel AR sites were proximal to genes containing ERG peaks, suggesting that ERG plays a direct and indirect role in AR cistrome reprogramming by tapping into a network of established enhancers (53). Additionally, ERG displayed an ability to significantly rescue the AR transcriptome following castration in mice prostate with *PTEN* deletion (53). In a separate study using organoids derived from the *PTEN* deletion and prostate-specific *ERG* overexpression mouse model described by Chen et al. (2013), ERG was shown to have permanent effects on AR cistrome reprogramming even after *ERG* knockout (54). However, AR transcriptional activity was significantly reduced with *ERG* deletion (54). These findings, coupled with evidence of ERG physically interacting with AR and AR coregulators (7, 54), suggest that ERG functions as a coactivator to AR in this setting, while simultaneously remodeling AR cistrome binding patterns through its pioneer factor activity (7, 54). Interestingly, in the Mao et al. (2019) study, ERG was found to play a crucial role in mediating resistance to Enzalutamide and PI3K pathway inhibition combination therapy in a *PTEN* deletion and prostate-specific *ERG* overexpression mouse model through a mechanism of ERG binding to AR target genes and maintaining their expression following AR inhibition (55). This activity occurred even in the absence of AR, with ERG promoting a luminal lineage within the tumors despite AR’s indifference (55). These findings contradict those of the Blee et al. (2018) study. However, a key difference between the two model systems is that the endogenous *TMPRSS2* promoter drives ERG in the Mao et al. (2019) study, which is less dependent on androgen activity and was also shown to be acted upon by ERG via ERG binding to an enhancer of *TMPRSS2* and driving its own expression (55). ERG’s action as a coactivator was further supported by *in vitro* DNA binding assays that revealed the ability of ERG protein to increase the binding affinity of AR protein, through a physical interaction between the AR LBD and the AR-interacting motif present in the ETS domain of ERG, to high-affinity (full) or low-affinity (half) ARE sequences (56). ERG was able to increase AR’s binding affinity to these sequences in the presence of AR-bound Enzalutamide (56), and the interaction was not dependent on ERG’s ability to bind to the DNA sequences, suggesting ERG can act as an AR coactivator without an ETS motif present (56). A subsequent structural study involved a NTD-truncated form of AR and full ERG protein combined *in vitro* with palindromic ARE DNA fragments; the resulting cryo-EM experiments revealed three DNA binding states, two of which are a “divorced” structure of two AR homodimers with limited interdomain contact, and an “entrenched” structure of two AR homodimers with unresolved electron density that could accommodate the ETS and PNT domains of ERG (57). Although a fully resolved cryo-EM structure of both AR and ERG was not yet solved, the existence of an ERG-AR complex was verified via XL-MS, SDS-PAGE, and immunoblotting experiments (57). Importantly, full-length AR transactivation assays in the presence of ERG showed that ERG resulted in a 20-fold increase of AR transactivation on a half ARE reporter versus a palindromic ARE sequence (57), while also increasing AR transactivation on a half ARE reporter when AR is bound to Enzalutamide versus DHT (57). However, ERG did not affect the transactivation of AR-v7 (57), which lacks the LBD, consistent with ERG’s physical interaction with AR occurring between the AR LBD and the ERG ETS domain (56). These findings suggest that ERG may best modulate AR, rather than AR-v7, when bound to DNA in a weaker, “divorced” state induced by half-ARE sites or allosteric Enzalutamide inhibition (56, 57).

CBP/p300 and ERG play critical roles in modulating the AR cistrome and regulating its transcriptional activity. p300 has been revealed to directly bind to both NTDs of an AR homodimer at ARE DNA through cryo-EM studies (44), while there is strong evidence for ERG binding at the LBD of AR in a similar resolved structure lacking the AR NTD (57). ERG is a nuanced partner of AR, acting as an AR corepressor in *PTEN*-positive PCa and as an AR coactivator in *PTEN*-null PCa. Furthermore, ERG has displayed pioneer-like activity by interacting with active enhancers to promote global AR binding; it also protects the AR cistrome from becoming indifferent in a *PTEN-null* context, preserving a luminal phenotype and resisting progression towards neuroendocrine prostate cancer (NEPC). Whether ERG plays a role in noncanonical AR cistrome reprogramming in EnzaR-CRPC remains to be determined; the role of CBP/p300 in supporting a noncanonical AR axis during EnzaR-CRPC will be discussed in later sections.

### 3.4 CREB5: a novel canonical AR cistrome coactivator and driver of enzalutamide resistance

CREB5 is a member of the cAMP response element binding protein (CREB) transcription factors, which bind to cAMP response elements (CREs) to activate transcription of target genes (58). CREB5 has been implicated in the progression of various cancers, including PCa (58). A 2019 study used an open-reading-frame screen in LNCaP cells exposed to hormone-stripped media and Enzalutamide to identify potential drivers of treatment resistance in PCa (59). This screen identified CREB5 as a potent contributor to castration and Enzalutamide resistance, with results being validated by CREB5 overexpression in several PCa cell lines *in vitro*, and *in vivo* with a CREB5-overexpressing LNCaP xenograft model in castrated mice (59). Importantly, the identified mechanism revealed that CREB5 directs AR to an alternative set of canonical AR target genes in a FOXA1-dependent manner (59). The transcriptional programs activated by the CREB5-AR axis reflected those observed in mCRPC patients, including MYC and cell cycle gene expression (59). In a subsequent study, rapid immunoprecipitation and mass spectrometry of endogenous proteins (RIME) in CREB5-overexpressing LNCaP cells exposed to Enzalutamide revealed that CREB5 physically interacts with AR, as well as with known AR cofactors and coactivators, such as FOXA1, HOXB13, and p300 (60). When analyzing ChIP-seq data from the same model system, CREB5 and FOXA1 cistromes were found to heavily overlap each other, with CREB5 and FOXA1 co-binding occurring at previously identified AR binding sites in mCRPC patients (11, 60). Furthermore, an integrative analysis of RIME findings and gene expression data from the SU2C mCRPC cohort (61) revealed that CREB5 expression correlated with the epithelial-mesenchymal transition (EMT) and β-catenin pathways, both of which are associated with resistance to AR-targeted therapies (60). As CREB5 expression was altered exogenously in these studies, it will be interesting to see whether these findings are further validated in a CRPC model system in which CREB5 is inherently upregulated.

Overall, CREB5 has been identified as a novel coactivator of AR in the context of Enzalutamide resistance, where it collaborates with the pioneer factor FOXA1 to redirect AR to canonical binding sites and to promote transcriptional programs that encourage treatment resistance and metastasis. Further studies using an EnzaR-CRPC model with endogenously elevated CREB5, as observed in some mCRPC patients, would strengthen these recent findings and provide greater insight into the potential for therapeutically targeting CREB5 in this setting.

### 3.5 CXXC5 and TET2

In a 2021 study aimed at uncovering AR-dependent mechanisms of resistance to Enzalutamide, Enza-R cell lines were generated from AR-positive CRPC models by continuous Enzalutamide treatment, with C4–2 Enza-R cells serving as the model of focus (62). Transcriptional analysis suggested that the proliferation of the Enza-R cells was driven by full-length AR independent of AR splice variants, with a downregulation of canonical AR gene targets (62). ChIP-seq experiments performed on C4–2 Enza-R and control cells revealed a set of AR binding sites (ARBS) gained in Enza-R cells compared to control, as well as sets of lost and unchanged ARBS (62). The gained ARBS were unique in that they showed enrichment in promoter and putative enhancer markers compared to lost or unchanged ARBS, indicating upregulation of transcriptional activity at these sites (62). However, the opposite trend was seen in enrichment of ARE or FOXA1 motifs at the gained ARBS, an interesting finding given that a previous study revealed that FOXA1 and HOXB13 pre-marked noncanonical AR signaling sites in primary PCa and CRPC (11).

**Figure 2:**
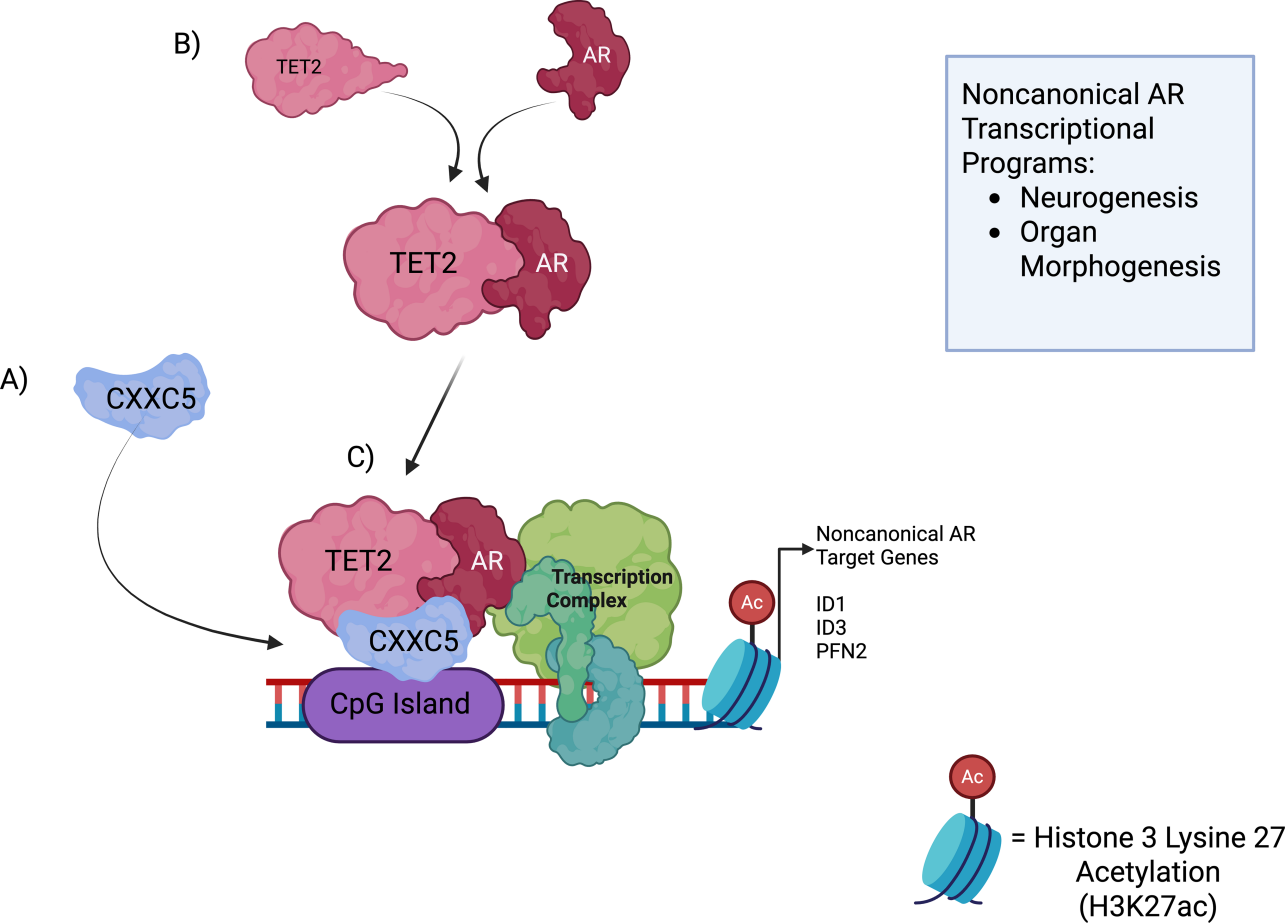
Mechanism for the formation of an AR-CXXC5-TET2 noncanonical complex. This figure is adapted from (29). **(A)** CXXC5 first binds to unmethylated, GC-rich regions at noncanonical genomic loci. These sites are flanked by H3K27ac, indicative of transcriptionally active chromatin. **(B)** TET2 and AR physically interact, which was observed in both CRPC and Enza-R CRPC conditions. **(C)** TET2, AR and CXXC5 form a noncanonical transcriptional complex, promoting expression of target genes that allow for lineage plasticity and differentiation potential, all of which contribute to Enzalutamide resistance. Figure created with BioRender.

As revealed by motif analysis, the gained ARBS showed enrichment for CpG islands (**Figure 2**), which are recognized by zinc-finger CXXC-domain-containing proteins (63). CpG islands are regions of GC-rich, unmethylated DNA that are present in 50-70% of vertebrate gene promoters (64, 65); 82% of the gained ARBS in the He et al. (2021) study contained CpG islands, suggesting this phenomenon is not due solely to the comparatively increased promoter presence at the gained ARBS (62). Out of 12 CXXC domain genes, *CXXC5* was found to be upregulated in gene expression and protein levels within the C4–2 Enza-R cells (62). The enzyme tet methylcytosine dioxygenase 2 (TET2), which contributes to DNA demethylation and requires binding to CXXC4 or CXXC5 to function (66–68), was also found to have elevated protein levels (62). Interestingly, TET2 has been shown to bind AR and several AR coactivators in hormone-sensitive prostate cancer (HSPC) LNCaP cells, where it represses transcription of a subset of canonical AR genes; loss of TET2 results in increased KLK3 gene expression and higher PSA protein levels (69). Using protein-binding assays, TET2 was shown to bind AR in both control and Enza-R conditions, whereas binding between CXXC5 and AR was observed only in Enza-R conditions (62). Downstream ChIP-seq analysis revealed enrichment of CXXC5 and TET2 co-bound with AR at noncanonical ARBS gained sites (**Figure 2**) independent of FOXA1 or ARE motifs (62).

Gene set enrichment analysis of RNA-seq data between C4–2 Enza-R and control cells revealed upregulation of genes crucial for lineage development and morphological changes, with the top genes including *ID1*, *ID3*, and *PFN2* (62). ID1, ID3, and PFN2 have been shown to be oncogenic factors that promote tumor cell lineage plasticity and proliferation (70–72). A previous study on the non-canonical AR cistrome found that the non-canonical AR signature is associated with a worse outcome in CRPC patients (12). Interestingly, analysis of RNA-seq data revealed that a high noncanonical AR signature correlated with a worse outcome for CRPC patients treated with AR signaling inhibitors; the same trend was observed with increased gene expression of *CXXC5* and the noncanonical target genes *ID1* and *PFN2* (62). The upregulation of CXXC5 coincides with findings from a previous study that documented CXXC5 mRNA and protein levels across normal prostate tissue and disease stages (73). In C4–2 Enza-R cells, increased expression of noncanonical AR target genes was confirmed, and knockdown of *AR, CXXC5, or TET2* reduced target expression (62). Additionally, knockdown of *CXXC5, TET2*, and the noncanonical targets *ID1, PFN2*, and *ID3* all reduced proliferation of Enza-R cells, underscoring their contributions to resistance (62). TET2 was found to play a key role in this mechanism of resistance, as its expression was required for AR residency at noncanonical gene promoters, and TET2 binding to CXXC5 was critical for maintaining noncanonical AR gene expression and Enza-R cell proliferation (62). The importance of TET2 and CXXC5 binding in noncanonical cistrome function is evidenced by the ability of rescued *CXXC5* to restore proliferation in *CXXC5* knockdown Enza-R cells. In contrast, rescue with a CXXC5 mutant unable to bind TET2 did not restore proliferation (62). These findings suggest that CXXC5 binds to CpG islands and provides a scaffold for TET2 and AR to bind (**Figure 2**) (62).

Overall, the study conducted by He et al. (2021) uncovered a novel mechanism of resistance to Enzalutamide by which, in the presence of repressed canonical AR signaling, TET2 associates with AR and the two proteins are recruited to CpG islands bound by CXXC5, thus resulting in AR binding at noncanonical genome loci (**Figure 2**) (62). Once intact, the AR-TET2-CXXC5 complex mediates transcription of noncanonical genes that allow for cellular lineage plasticity and enable PCa cells to proliferate in an AR-dependent, ARE/FOX-independent manner (62).

### 3.6 EZH2

The epigenetic regulator EZH2 has been shown to be upregulated during PCa progression, with increased expression correlating with poorer patient prognosis (74). In PCa, EZH2 appears to play an oncogenic role in cancer progression and treatment resistance through multiple mechanisms (75). EZH2 is known to canonically associate with Suppressor of Zeste 12 Protein Homolog (SUZ12) and Embryonic Ectoderm Development (EED) to form a Polycomb Repressive Complex 2 (PRC2) complex; in this complex, EZH2 catalyzes histone 3 lysine 27 trimethylation (H3K27me3), contributing to gene silencing and transcriptional repression (75). EZH2 was found to stabilize the pioneer factor FOXA1 through its methylating activity in a Polycomb-dependent mechanism, resulting in co-regulation of genes involved in cell proliferation and cell cycle progression (76). EZH2 has also been found to interact with AR through several pathways, including as a coactivator to AR in CRPC and as an activator of AR gene transcription in primary PCa and CRPC; both activities are PRC2-independent (77–79). Interestingly, EZH2, along with ERG and HDAC1-3, was discovered to act as a corepressor of AR in androgen sensitive VCaP cells, resulting in reduced transcription of canonical AR target genes such as *KLK3* (PSA) and *FKBP5* (51); EZH2 and its collaborating corepressors also suppressed transcription of the AR target Vinculin (*VCL*) in an effort to promote progression towards metastasis (51).

**Figure 3:**
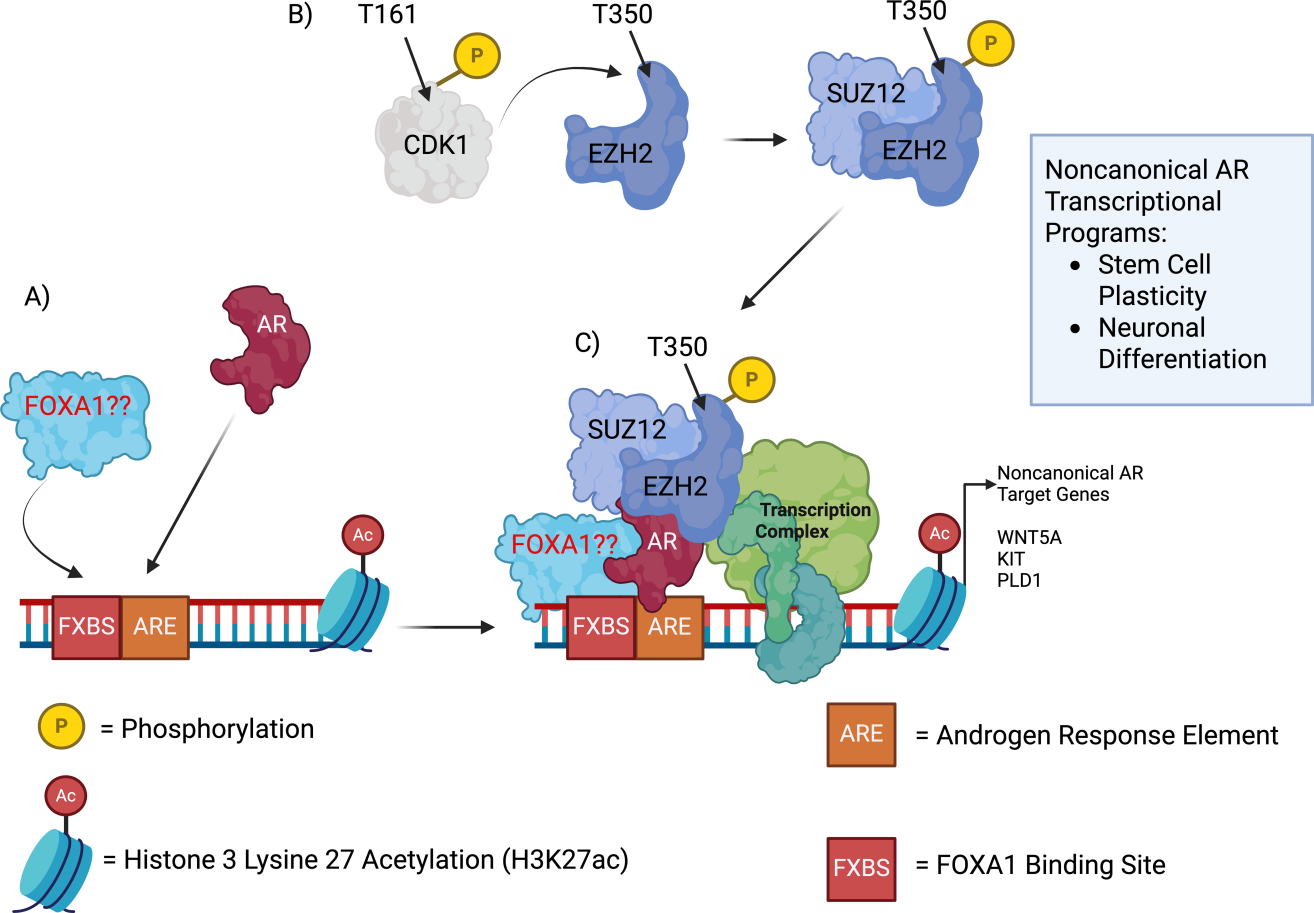
AR-EZH2 noncanonical complex formation in Enza-R CRPC. **(A)** AR was found to bind at half androgen response element (hARE) sites along with FOXA1 binding sites; these sites are flanked by regions of H3K27ac, indicating increased transcriptional activity. While AR and FOXA1 are confirmed to bind via RIME in Enza-R CRPC, it remains to be seen whether FOXA1 binds to these noncanonical AR binding sites or acts as a pioneer factor to make them accessible to AR. **(B)** CDK1 in its activated form (phosphorylated at residue T161) phosphorylates EZH2 at residue T350; this activated form of EZH2 was found to be the driver (along with AR) of lineage plasticity in Enza-R CRPC. EZH2 forms a “noncanonical” PCR2 complex with SUZ12. **(C)** The noncanonical AR complex consists of AR bound to activated EZH2 and/or AR bound to EZH2 and SUZ12, though to a lesser degree. These noncanonical AR transcriptional complexes promote transcription of target genes associated with stemness and lineage plasticity, enabling resistance to Enzalutamide pressure. Figure created with BioRender.

A recent study by Davies et al. (2021) uncovered a novel role for EZH2 in mediating a noncanonical AR cistrome switch in Enza-R CRPC, resulting in cell lineage plasticity (80). The authors utilized an approach highlighted in a previous study (81) to generate Enza-R models *in vivo* from CRPC (16D CRPC), resulting in 42D Enza-R, a model of AR+ and PSA- cells with a loss of canonical AR signaling (80). 42D Enza-R displayed AR cistrome reprogramming when compared to 16D CRPC, with an enrichment of binding at half androgen response elements (hARE) and FOXA1 binding sites (FXBS) (**Figure 3**) (80). The AR binding pattern of 42D Enza-R was consistent with a loss of canonical AR signaling, but an increase in noncanonical binding at genes involved in neuronal development; these noncanonical cistrome sites overlapped with those identified in Enza-R patient tumors (80). RNA-seq data from 42D Enza-R provided further evidence towards the switch from canonical AR genes to neuronal and stem cell programs in 42D Enza-R (80).

To identify factors mediating the noncanonical AR cistrome switch, AR RIME was performed in 16D CPRC and 42D Enza-R cells; SUZ12 and EED, members of the PRC2 complex, were pulled down with AR at a higher frequency in 42D Enza-R than in 16D CPRC (80). SUZ12 Co-IP confirmed an interaction with AR and EED, while AR and EZH2 were found to interact in the nucleus of 42D Enza-R cells via proximity ligation assay (PLA) (80). Analysis of the cistromes of each PRC2 subunit in 42D Enza-R revealed an enrichment of AR peaks associated with EZH2 alone (AR-EZH2), or with EZH2 and SUZ12 to a lesser extent (**Figure 3**) (80). H3K27ac and ATAC signals had increased overlap at AR-EZH2 peaks, indicating active transcription at these shared sites (**Figure 3**) (80).

With deletion of *AR* or EZH2 inhibition, AR-EZH2-associated genes such as *WNT5A*, *KIT*, and *PLD1* were subsequently downregulated in 42D Enza-R (80). *WNT5A*, *KIT*, and *PLD1* are noted oncogenes that promote cancer stemness, differentiation, migration/invasion, and chemoresistance (82–85). Of note, EZH2 inhibition was sufficient to displace EZH2, but not AR, from co-bound regions, which may indicate that AR initially binds at these noncanonical sites and then recruits EZH2 (**Figure 3**) (80). These findings revealed a noncanonical interaction between AR and EZH2 at reprogrammed AR genome loci in Enza-R PCa, where both players mediate transcription of genes involved in neuronal development and stem cell plasticity. Based on these discoveries, the authors determined whether EZH2 is necessary for the switch from CRPC to the neuroendocrine-like Enza-R CRPC observed in the study. After CRISPR-Cas9 deletion of *EZH2* in 16D CRPC and following Enza treatment, expression of neuroendocrine genes was ablated compared to control, and CPRC, *EZH2-*deleted xenografts failed to grow with Enza treatment (80). This mirrored a previous study where knockdown of *EZH2* sensitized Enza-R CRPC xenografts to Enza treatment, resulting in reduced cell proliferation and increased apoptosis (86).

The authors then focused on post-translational modifications (PTMs) of EZH2 to examine how they might contribute to Enzalutamide resistance. While EZH2 S21 phosphorylation (pS21) is required for AR activation in CRPC (77), this PTM was not significantly upregulated in 42D Enza-R cells or in neuroendocrine patient tumors compared to CRPC (80). However, phosphorylation of T350 (pEZH2-T350) was found to be enriched in the same settings (80). CDK1 is known to phosphorylate EZH2 at T350 (**Figure 3**) (87); this relationship was evident, as activated CDK1 (pCDK1-T161) was upregulated along with pEZH2-T350, whereas CDK1 inhibition ablated pEZH2-T350 levels and reduced target gene expression (80). The pEZH2-T350 RIME showed a strong association with SUZ12 (**Figure 3**), mirroring the AR RIME findings (80). 42D Enza-R *EZH2* KO cells expressing a phosphomimetic mutant (EZH2-T350D) had enriched gene sets for chromatin remodeling, neural stem cell differentiation, and plasticity (mirroring findings in EZH2-high, neuroendocrine patient tumors). In contrast, non-phosphorylating EZH2 mutants (EZH2-T350A) in the same *EZH2* KO background showed downregulation of those gene pathways (80). Through these findings and others, the authors uncovered a role for pEZH2-T350 in promoting cell lineage plasticity of CRPC to evade Enzalutamide.

Whether the effects of pEZH2-T350 were seen in tandem with AR remained to be seen. ChIP-seq analysis in 42D Enza-R cells revealed noncanonical AR co-binding at EZH2 and pEZH2-T350 peaks, with an enriched overlap of AR-pEZH2-T350 with SUZ12 (**Figure 3**) compared to EED (80). Importantly, genes with promoters co-bound by AR and pEZH2-T350 showed active expression in 42D Enza-R cells, and these same genes showed increased expression in patient tumors following Enzalutamide treatment. They were enriched for stem cell and plasticity transcriptional programs (80). As AR and EZH2 shared the ability to modulate cell lineage in resistance to Enzalutamide, the researchers sought to interrogate the effects of inhibiting AR or EZH2 on cell-fate direction. *AR* KO in 42D Enza-R cells increased NEPC transcriptional score, with gene sets highly enriched for neuronal growth and signaling (80). In contrast, EZH2 inhibition of 42D Enza-R cells with the compound GSK126 activated canonical AR signaling and altered the transcriptome to resemble AR-driven CRPC cells (80) more closely. EZH2 inhibition, but not EED inhibition, disrupted AR-EZH2 interactions and decreased SUZ12 chromatin binding, thereby physically preventing AR-EZH2 or AR-EZH2-SUZ12-associated noncanonical AR signaling (80). In addition, EZH2 inhibition resensitized 42D Enza-R cells to Enzalutamide (**Figure 3**); this anti-proliferative effect was reversed with EZH2 inhibitor washout, resulting in the return of 42D Enza-R cells to a lineage-plastic state (80).

By summing these findings, the Davies et al. (2021) study revealed a novel role for EZH2 as a coactivator of AR in the context of Enzalutamide resistance, resulting in AR cistrome reprogramming and noncanonical AR binding to genes critical to neuronal development and lineage plasticity. SUZ12 was also found to be associated with both EZH2 and AR at reprogrammed genome loci, suggesting AR-EZH2 or AR-EZH2-SUZ12 exist in “noncanonical PCR2 subcomplexes” at these sites (**Figure 3**) (80). The activity of EZH2 in promoting a lineage plasticity state was found to be driven by phosphorylation of T350 by activated CDK1, and the pEZH2-T350 was enriched at non-canonical AR binding sites and associated with transcriptionally active chromatin (80). pEZH2-T350 was later determined to be required for the development and maintenance of a neuroendocrine-like, cellular plasticity state (80). The researchers discovered that the noncanonical AR-EZH2 binding resulted in a “lineage-infidelity” state of Enzalutamide-resistant PCa, one that could lead to reversion to canonical AR signaling or differentiation into NEPC with suppression of EZH2 or AR, respectively (80).

## 4 Implications of noncanonical AR coactivators

The two cases covered here, which outline noncanonical AR cistrome-dependent mechanisms of resistance to Enzalutamide treatment, raise important questions about lineage plasticity, epigenetic modifications, and chromatin remodeling at this stage of PCa. It is poignant that the three noncanonical AR coactivators that have been recently discovered all have been known to play some role in the regulation of epigenetic markers or gene expression. Interestingly, TET2, an enzyme that catalyzes DNA demethylation, is a tumor suppressor in prostate cancer and is frequently mutated, with loss of TET2 expression associated with cancer progression and reduced patient survival (69, 88). The opposite trend of TET2 expression was observed in the He et al. (2021) study, where TET2 protein was lowly expressed in CPRC but upregulated in Enza-R CRPC (62). TET2 lacks a CXXC domain and thus relies on interactions with CXXC4 or CXXC5 to bind to DNA (**Figure 2**); however, the demethylation function of TET2 is associated with its catalytic domain and occurs in a Fe (II), 2-oxoglutarate (2-OG)- dependent manner (67, 68). In the He et al. (2021) study, CXXC4 was not readily expressed in C4–2 Enza-R cells; a previous study revealed that CXXC4 and CXXC5 interactions with TET2 may serve to regulate TET2 expression by mediating its degradation, though the findings were observed in HEK293T cells that did not naturally co-express both proteins (62, 67, 89). In the Ma et al. (2017) study, the interaction between CXXC5 and TET2 led to hypomethylation of CpG islands and subsequent active transcription at a subset of genes required for an anti-viral interferon (IFN) immune response in plasmacytoid dendritic cells (68). Perhaps a similar mechanism occurred in the He et al. (2021) study between CXXC5 and TET2 to modulate gene expression and to reveal access to gene promoters, where the additional interactions between TET2 and AR enabled AR binding to demethylated CpG islands at noncanonical gene targets, promoting resistance to Enzalutamide (**Figure 2**). Interestingly, CXXC5 has been shown to activate transcription of myelin genes during oligodendrocyte differentiation (90), which aligns with the shift towards a neuroendocrine-like state observed in Enza-R CRPC. The findings of the He et al. (2021) study identify CXXC5 and TET2 as potential biomarkers of noncanonical AR Enzalutamide resistance. However, direct therapeutic inhibition of these two transcription factors has not yet been explored.

While AR was found to bind to noncanonical sites at CpG island motifs in the He et al. (2021) study, in the Davies et al. (2021) study, AR was observed to colocalize with EZH2 at noncanonical sites, which contained hARE/FOX motifs (**Figure 3**) (80). AR RIME of 42D Enza-R also revealed FOXA1 as the most enriched binding partner, more so than the PRC2 subunit SUZ12 (80). However, FOXA1 ChIP-seq was not performed in this study, so we cannot yet confirm whether FOXA1 co-localizes with AR and EZH2 as part of the uncovered noncanonical AR-binding complex (**Figure 3**). This could be an important finding, as FOXA1 is a well-known AR collaborator and driver of PCa, and AR cistrome reprogramming; any role of FOXA1 in the noncanonical AR cistrome in response to AR-targeted therapies has yet to be identified. Given FOXA1’s known function as a pioneer factor, it would be interesting to determine whether FOXA1 plays a role in binding and “pre-marking” these Enza-R CRPC noncanonical binding sites before AR binds (**Figure 3**), as was observed previously in the transition from primary PCa to mCRPC (11). The He et al. (2021) and Davies et al. (2021) studies revealed that noncanonical AR cistrome reprogramming in Enza-R CRPC resulted in gene programs associated with lineage plasticity or neuroendocrine differentiation. As previously mentioned, Class 2-mutated FOXA1 in mice prostate displayed the ability to generate a stem-like cell population that was resistant to castration and Enzalutamide, though in an AR-independent manner, as the mutant FOXA1-AR co-bound sites remained largely unchanged compared to WT FOXA1 (35). Further understanding the contributions of FOXA1 could provide additional insight into cistrome reprogramming or chromatin remodeling within these cellular states, particularly in the mechanism of AR-pEZH2-T350 driven “lineage-infidelity”, as it has been recently found that both EZH2 and FOXA1 are involved in the transition of Enza-R CPRC to NEPC (91–93). The Davies et al. study uncovered further evidence in the involvement of EZH2 with NEPC transition, as functional RB1 loss, which, along with TP53 loss, is essential to NEPC progression (93), causes upregulation of pEZH2-T350 in organoids derived from a NEPC PDX model (80). The role of EZH2 in stabilizing FOXA1 (76) may provide further evidence of potential FOXA1 involvement in EZH2-dependent, noncanonical AR cistrome reprogramming. Another question that would follow if FOXA1 is localized with AR and EZH2 is whether the SWI/SNF complex is involved in promoting noncanonical AR at a lineage-plastic state, which would contrast the findings from Gokbayrak et al. (2025) (24). This may not be possible as SWI/SNF and Polycomb complexes often have antagonistic functions (21), though a non-canonical Polycomb complex exists in this model of EnzaR-CRPC that activates, rather than represses, a non-canonical AR cistrome.

## 5 Treatment of noncanonical AR cistrome-driven Enza-R CRPC

**Figure 4:**
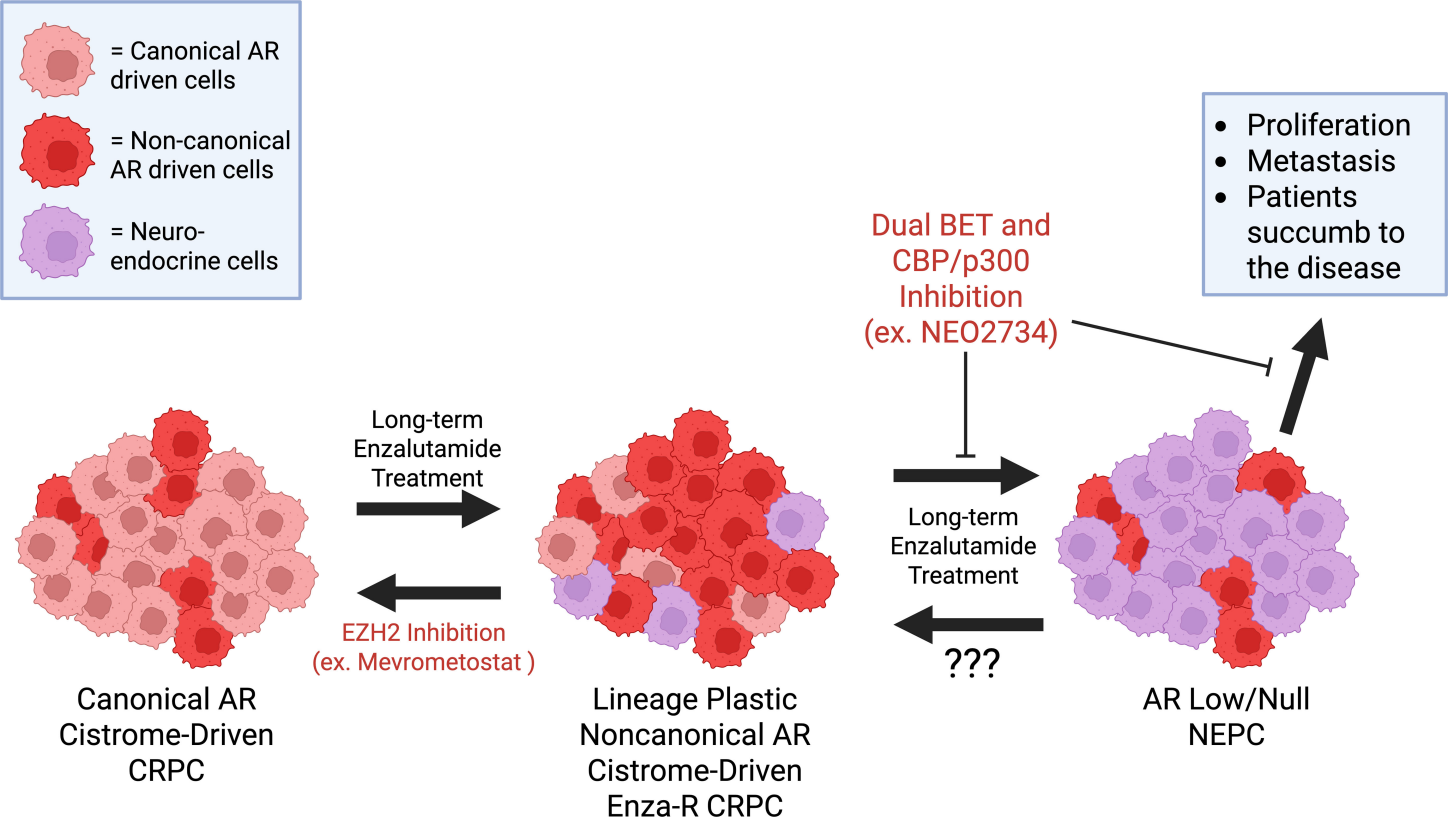
Potential treatment strategy for patients who have noncanonical AR-driven Enza-R CRPC. This model accounts for PCa heterogeneity, recognizing that multiple cell lineages can coexist within a patient’s tumor at different stages of disease. He et al. (2021) reported that dual inhibition of BET and CBP/p300 proteins is sufficient to disrupt the AR-CXXC5-TET2 noncanonical signaling axis, resulting in an antitumor effect and potentially preventing the progression of Enza-R CRPC to NEPC. Davies et al. (2021) showed that EZH2 inhibition reverses the AR cistrome toward a canonical cistrome program seen in CRPC, thereby resensitizing the cancer cells to Enzalutamide treatment. Both dual BET and CBP/p300 inhibitors and EZH2 inhibitors are currently in human clinical trials for patients with advanced PCa. Figure created using BioRender.

As advanced PCa ultimately proves fatal for many patients who have exhausted current treatment modalities, preclinical studies must help to uncover novel therapeutic strategies for these patients. Such strategies may include pharmacological targeting of noncanonical AR axes or pathways triggered by noncanonical AR cistrome reprogramming. Prior studies using noncanonical AR-driven EnzaR-CRPC models found that blockade of the PI3K/AKT pathway with Ipatasertib led to repression of GR, increased canonical AR signaling, and subsequent re-sensitization of Enza-R CRPC to Enzalutamide (6). Fortunately, the He et al. (2021) and Davies et al. (2021) research groups tested inhibitors against the noncanonical AR axes identified in their respective studies. In the He et al. study, the compound NEO2734, a dual inhibitor of BET and CBP/p300 proteins, was found to inhibit cell growth in C4–2 Enza-R cells and Enza-R PDX models via reduced expression of AR and CXXC5, and reduced occupancy of CXXC5, TET2, and AR proteins at noncanonical AR target gene loci (**Figure 4**) (62). BET proteins, such as BRD4, are known AR coregulators, and CBP/p300 are known AR coactivators (17, 27, 30, 41, 94). BET and CBP/p300 increase transcriptional activity and are critical drivers of chromatin remodeling, a mechanism that may enable noncanonical AR cistrome reprogramming (17, 30, 31, 40, 94, 95). Apart from the He et al. (2021) study, NEO2734 has displayed antitumor effects in several studies involving models of prostate cancer, lymphoma, and leukemia (95–97). As a result, NEO2734 has been monitored in a Phase I human clinical trial for patients with CRPC and hematological malignancies since 2022, with the study expected to end in May 2025; the last trial update was posted in January 2025, while no new updates on trial progress have been posted as of December 2025. (**Table 1**) (NCT05488548). While BET and CBP/p300 are widely expressed across tissues and regulate the activity of a diverse group of transcription factors (17, 96), the effectiveness of NEO2734 against Enza-R CRPC could indicate an interaction of BET and CBP/p300 with AR specifically at noncanonical AR cistrome foci (62). This may present a viable treatment option for patients who are found to have CXXC5-dependent, noncanonical AR signaling as an Enzalutamide resistance mechanism (**Figure 4**). Interestingly, NEO2734 reduced proliferation of AR null, NEPC models *in vitro* and *in vivo*, while simultaneously suppressing transcription of neuroendocrine markers ASCL1 and SYP (96). This therapeutic approach did not result in the re-expression of AR or other canonical AR genes, aside from modest NKX3.1 upregulation, suggesting NEO2734 may slow progression of NEPC (**Figure 4**) but may not be sufficient to revert NEPC to an adenocarcinoma state (96). Another intriguing thought is whether p300 inhibition may alter potential SWI/SNF binding at these non-canonical loci, since SWI/SNF complexes can recruit p300 (21); however, SWI/SNF presence at these loci would need to be confirmed through further investigation.

**Table 1:** List of noncanonical AR axis inhibitors and current clinical trial information.

Inhibitor name	Target	Clinical trial ID	Indication in clinical trial	Current status of clinical trial
NEO2734 (or EP31670)	Dual inhibitor of BET and CBP/p300 proteins	NCT05488548	Castration-resistant prostate cancer, other advanced solid tumors, chronic myelomonocytic leukemia, myelofibrosis, other hematological malignancies	Actively recruiting/monitoring patients in Phase I of the clinical trial, expected to end around May 2025 (no trial update posted as of November 2025)
GSK126	EZH2 inhibitor	NCT02082977	Castration-resistant prostate cancer and other solid tumors, relapsed/refractory diffuse large B cell lymphoma, transformed follicular lymphoma, other Non-Hodgkin’s lymphomas, multiple myeloma	Study terminated after completion of Phase I due to insignificant clinical effect and dose-limiting toxicity
CPI-1205	EZH2 inhibitor	NCT03480646	CPI-1205 in combination with either Enzalutamide or Abiraterone/Prednisone in patients with metastatic Castration Resistant Prostate Cancer	Study initially discontinued during Phase I/II after data revealed poor efficacy of treatment; study was amended and continued, status on clinical trial website reads as completed as of 10/29/25
Tazemetostat	EZH2 inhibitor	NCT04179864	Tazemetostat in combination with either Enzalutamide or Abiraterone/Prednisone vs. Enzalutamide or Abiraterone/Prednisone alone in patients with metastatic Castration-Resistant Prostate Cancer	Study terminated at Phase Ib/II due to insignificant benefit in combination treatment compared to Enzalutamide alone
Tulmimetostat (CPI-0209)	EZH2 inhibitor	NCT04104776	Patients with advanced solid tumors and lymphomas, including patients in cohort M6 (metastatic Castration-Resistant Prostate Cancer, mCRPC)	Study is still active in both Phase I and Phase II (the M6 mCRPC cohort was initially discontinued in preliminary Phase II after a 0% objective response rate was observed, but has since continued) and recruiting as of 10/15/25; planned completion date is 2030
PF-06821497 (Mevrometostat)	EZH2 inhibitor	NCT03460977 NCT06551324	Mevrometostat administered as a single agent or in combination with standard of care (SOC) to patients with Relapsed/Refractory Small Cell Lung Cancer, Castration Resistant Prostate Cancer (CRPC, SOC is Enzalutamide), and Follicular Lymphoma Mevrometostat in combination with Enzalutamide vs. physician’s choice of Enzalutamide or Docetaxel (both SOC) in patients with metastatic Castration Resistant Prostate Cancer	In the Phase I clinical trial, Mevrometostat showed promise in combination with Enzalutamide in CRPC patients who had previously received Enzalutamide or Abiraterone, and the safety profile is reasonable A new Phase III trial was initiated in October 2024 to determine the safety and efficacy of Mevrometostat and Enzalutamide combination vs. SOC alone in metastatic CRPC patients. Study is currently recruiting patients
SHR2554	EZH2 inhibitor	NCT03741712 NCT06568094	SHR2554 alone or in combination with SHR3680 (a novel AR-antagonist) in patients with metastatic Castration Resistant Prostate Cancer SHR2554 in combination with HRS-5041 (a novel AR-antagonist) in patients with advanced Prostate Cancer	Study terminated during Phase I/II due to sponsor decision after reviewing available data from the trial SHR2554 is being investigated in combination with a novel AR-antagonist in a new Phase I/II clinical trial which was initiated in September 2024. Study is currently recruiting patients

As EZH2 was identified in the Davies et al. (2021) study as a crucial driver of the noncanonical AR cistrome and the “lineage-infidelity” state that exists between CRPC and NEPC, targeting EZH2 may represent a viable treatment strategy for advanced PCa patients with resistance to AR-targeted therapies (**Figure 4**). In the Davies et al. (2021) study, inhibition of EZH2 with GSK126 led to 42D Enza-R cells reverting to canonical AR signaling, thereby resensitizing them to Enzalutamide treatment (**Figure 4**) (80). Interestingly, a study using a triple-knockout mouse model of NEPC (*PTEN*, *RB1*, and *TP53* null) showed that inhibition of EZH2 leads to upregulation of AR gene and protein levels and subsequent canonical target gene expression, with concomitant Enzalutamide re-sensitization (**Figure 4**) (98). While several EZH2 inhibitors are currently undergoing or have recently completed clinical trials in metastatic CRPC patients, results have been mixed (**Table 1**) (93). GSK126, highlighted in the Davies et al. (2021) study, was terminated after its Phase I clinical trial, which showed an insignificant clinical effect and dose-limiting toxicity in patients with CRPC or hematological cancers (NCT02082977) (99). In clinical trials featuring CRPC or mCRPC patients, various other EZH2 inhibitors, including CPI-1205 and Tazemetostat, were discontinued due to poor efficacy (NCT04179864) (100). The CPI-1205 clinical trial was initially discontinued in Phase I/II, but after amendments, the trial was continued and completed in October 2025 (NCT03480646). The inhibitor Tulmimetostat (CPI-0209) is in an active Phase II clinical trial, but the mCRPC cohort was initially not expanded past preliminary Phase II due to a 0% objective response rate (NCT04104776) (101). Recent updates for CPI-0209 report that the mCRPC cohort has since continued, and the study is actively recruiting patients as of October 2025, with an expected completion date around 2030. After promising Phase I results, the compound PF-06821497 (Mevrometostat) has been advanced to Phase III of its clinical trial, where it is being paired with Enzalutamide vs. Enzalutamide or Docetaxel alone in mCRPC patients who previously progressed after abiraterone acetate treatment (NCT03460977, NCT06551324) (102). Another EZH2 inhibitor, SHR2554, was previously part of a Phase I/II clinical trial for mCRPC patients that was terminated by the sponsor (NCT03741712); it is now being investigated in combination with a novel AR antagonist (HRS-5041) in patients with advanced prostate cancer (NCT03741712, NCT06568094). There have also been several proteolysis-targeting chimeras (PROTACs) designed to target EZH2, though there is currently no clinical trial evaluating an EZH2 degrader in advanced PCa. The currently developed EZH2 PROTAC degraders have been extensively reviewed by Guo et al. (2024) (103). Overall, these recent findings reveal promise for the use of EZH2 inhibitors in noncanonical AR cistrome-dependent Enza-R CRPC. However, it remains to be seen whether an EZH2 inhibitor will be approved for use in AR-targeted therapy-resistant mCRPC. Preclinical findings suggest a potential therapeutic role for EZH2 inhibition in NEPC with low or null AR expression, though, at present, there are no clinical trials of EZH2 inhibitors involving NEPC patients.

## 6 Future directions in AR coactivator discovery

Advances in techniques and data analysis may be the key to uncovering additional trends in noncanonical AR cistrome reprogramming at the Enza-R CRPC stage, while also being used to identify novel AR coactivators, as highlighted in this review. One such technique is single-cell sequencing, which includes single RNA (scRNA-seq) and single-cell ATAC (scATAC-seq) sequencing assays (104). As the name suggests, these assays allow transcriptomic and epigenomic information to be obtained from a single cell, indicating cell state and cell type (104). Cells with correlated gene expression or chromatin signatures can be clustered together into populations via multimodal integration; these clusters can give insight into the heterogeneity of prostate cancer and how cell populations shift across disease stages (104). Two recent studies (105, 106) have employed this approach, using the well-characterized PCa model LNCaP, a hormone-sensitive cell line that can be made resistant to Enzalutamide to study PCa disease progression (107–110). The Taavitsainen et al. study compared LNCaP cells exposed to Enzalutamide for short-term treatment vs. Enzalutamide-resistant and RD-162 (a second-generation AR-antagonist)-resistant LNCaP cells at the single-cell level to better understand the dynamics of treatment resistance in PCa patients (105). LNCaP cells resistant to long-term AR-antagonist exposure were found to cluster in populations with distinct chromatin states and transcriptional patterns defined by differentially expressed genes; this analysis was made possible by label transfer of the integrated scRNA-seq data with the scATAC-seq data (105). One downside of the label transfer approach, however, is increased variation due to different sets of cells being compared. Interestingly, cells in Cluster 11 of the integrated scRNA-seq dataset remained in a persistent transcriptional state prior to and throughout the development of resistance; these cells were identified as having a “Persist” signature and expressed features of stemness, chromatin remodeling, and active cell cycling (105). Cluster 11 was found to have higher differentiation potential than the other clusters, suggesting that the other clusters may arise from persistent cells throughout the course of treatment (105).

The Asberry et al. study used a combined approach of bulk RNA-seq, CUT&RUN, and scRNA-seq to track epigenomic and transcriptional differentiation of LNCaP cells toward a neuroendocrine state (106). By comparing LNCaP cells exposed to 5 μM Enzalutamide for 4, 7, and 14 days, the researchers identified neuroendocrine-like (NEL) morphological changes that occurred in 80% of cells by Day 14. At the same time, transcription of neuroendocrine genes such as ASCL1 and CD56 increased over the course of treatment (106). During Enzalutamide treatment, scRNA-seq analysis identified a subsequent decline in clusters of cells with active cell cycling and canonical AR gene signatures. In contrast, clusters C2 and C5 showed NEL gene expression and were enriched by day 14 of treatment (106). However, AR expression, which increased across the dataset with treatment duration, was heterogeneously expressed in C2 and C5, while noncanonical AR signaling also increased as treatment progressed (**Figure 4**) (106). This discovery could have been strengthened through an assessment of AR binding patterns. These findings provide additional evidence for the existence of cells in a lineage-plastic state, displaying neuroendocrine differentiation while retaining functional noncanonical AR activity (62, 80, 106).

The single-cell analysis method provides greater depth of understanding of how treatment resistance in prostate cancer arises heterogeneously, suggesting that multiple cell populations in patients may be responsible for poor response (**Figure 4**) (105, 106). In the context of noncanonical AR cistrome in EnzaR-CRPC, these tools can be leveraged to identify novel AR coactivators by honing in on clusters of cells enriched for noncanonical AR activity and further investigating transcription factors that are found to drive the noncanonical signature along with AR, through such methods as differential gene expression analysis and transcription factor binding motif analysis of integrated scRNA-seq and scATAC-seq datasets (104). One such strategy would first be to use *de novo* TF motif discovery on AR ChIP-seq datasets, focusing on differentially expressed, noncanonical AR-binding peak regions, as performed in the He et al. and Davies et al. studies (62, 80). *De novo* TF motif discovery relies on computational models, such as the widely used position weight matrices (PWMs) (111–113). A variety of publicly available computational tools are available for *de novo* TF motif discovery; many of these have been thoroughly discussed in the Boeva et al. (2026) review (111). However, while PWMs tend to perform well for many TFs, it is limited by its assumption that each nucleotide within the motif is statistically independent, whereas biologically, DNA conformations are influenced by multiple consecutive stretches of nucleotides (111, 114). Higher-order models, such as Bayesian Markov models (BaMM), have been shown to outperform PWM-based models in predicting TF binding motif sequences across hundreds of ChIP-seq datasets (114, 115). Both PWM- and BaMM-based tools often include databases of known TF binding motifs, allowing active noncanonical AR cistrome loci to be scanned for TFs with established impact on AR binding or to uncover unknown AR collaborators (113). Overall, PWM- and BaMM-based computational tools can be leveraged to identify potential novel AR cofactors, discover alternative AR binding motifs, or to predict known TFs at noncanonical AR cistrome loci at the EnzaR-CRPC stage of PCa. Another approach would be to identify *de novo* TF motifs from genome-wide chromatin accessibility assays such as ATAC-seq (116). This can be achieved by exploiting “footprint” regions with reduced read signal, which may represent TF binding within an active chromatin region (116). While these “footprints” may map to known TF motifs, a recently described tool named DENIS (**DE N**ovo mot**I**f di**S**covery) (116) has been shown to derive *de novo* TF motifs from footprint regions, and cross-reference these motifs to ensure they are indeed novel (116). Protein binding partners for *de novo* TF motifs can then be discovered using a DNA-binding mass spectrometry approach, in which the newly identified DNA sequences are incubated with a cell lysate (117). Furthermore, a scATAC-seq dataset, such as that generated by Taavitsainen et al. (2021) (105), could be analyzed with the DENIS tool to identify *de novo* TF motifs enriched within cell clusters. The noncanonical AR cistrome regions identified from AR ChIP-seq studies can be overlapped with ATAC-seq datasets and further stratified by cell cluster to potentially reveal multiple noncanonical AR-driven clusters within an EnzaR-CRPC tumor, while highlighting AR coactivator candidates that drive the observed cistrome changes.

The discovery of AR cofactors and mediators of noncanonical AR cistrome is critical to identifying therapeutic targets for patients with advanced disease. With increasing development of high-throughput sequencing and bioinformatic approaches, powerful tools are now available to survey the AR cistrome within advanced, treatment-resistant PCa models at the bulk and single-cell level. Identified genomic regions of noncanonical AR activity can be probed to uncover *de novo* TF motifs, paving the way for mass spectrometry to identify these unknown AR cofactors. Furthermore, noncanonical AR cistrome regions can be compared across sequencing experiments (ChIP-seq vs. ATAC-seq) to identify known TFs that may be interacting with AR and drive this form of treatment resistance. Both known and unknown TFs discovered to collaborate with AR may represent new pharmacological targets, as the landscape of TF inhibition in the translational and clinical settings has grown in recent years (15).

## 7 Conclusions

Collaborators of the transcription factor AR have been found to modulate the AR cistrome, both in the normal prostate and at each stage of prostate cancer progression. As CRPC patients develop resistance to the AR antagonist Enzalutamide, AR activity has been observed to dissipate in patients who develop NEPC. Whether traditional AR coactivators such as HOXB13, GATA2, or ERG play a critical role in AR cistrome programming at the Enza-R CRPC stage remains to be seen; however, novel AR cistrome coactivators at this stage have been identified. The transcription factor CREB5 has been shown to drive canonical AR cistrome in Enza-R CRPC in a FOXA1-dependent manner. CXXC5, TET2, and EZH2 have been uncovered as novel co-activators of AR in the context of Enza-R CRPC, mediating noncanonical AR cistrome reprogramming as a mechanism of Enzalutamide resistance, resulting in lineage plasticity or neuroendocrine-like state dependent on noncanonical AR signaling. The AR-CXXC5-TET2 noncanonical axis is further supported by AR coregulators CBP/p300 and BET proteins. CXXC5, TET2, and EZH2 represent therapeutic targets for patients who are not responding to AR-targeted treatments and who are found to be in the intermediate “lineage-infidelity” state between CRPC and NEPC. These findings can further inform treatment strategies for patients with advanced, treatment-resistant prostate cancer, hopefully resulting in improved patient outcomes. Technological advances in genomic and epigenomic sequencing can be leveraged to uncover more novel AR coactivators, even potentially associating these coactivators with specific cell types within a tumor. Through multiomic approaches, the oncogenic transcription factor EVI1, encoded by the *MECOM* locus, was identified as a novel non-canonical AR coactivator (118). *MECOM* is upregulated in CRPC and EnzaR-CRPC models, and EVI1 and AR physically interact in EnzaR-CRPC models (118). Importantly, knockdown of *MECOM* reduced proliferation in 2D and 3D EnzaR-CRPC culture models and altered non-canonical AR signatures (118). As in the aforementioned LNCaP single-cell studies, we have taken a similar approach in single-cell RNA- and ATAC-seq analysis to further investigate populations that comprise CRPC and EnzaR-CRPC PDX models to identify drivers of noncanonical AR cistrome (119, 120); this approach, along with core regulatory circuitry analysis in EnzaR-CRPC cell lines, has identified PBX1 as a potential non-canonical AR cofactor, and pharmacologic inhibition of PBX1 in EnzaR-CRPC models has resulted in a significant antiproliferative effect (120). Hopefully, novel AR coactivators and drivers of the noncanonical AR cistrome may be the key to slowing or reversing disease progression in patients with Enzalutamide-resistant CRPC.
